# Supplementary material for: Quantum-mechanical machinery for rational decision-making in classical guessing game
Source: Sci Rep. 2016 Feb 15;6:21424. doi: 10.1038/srep21424 (PMC4753459; doi:10.1038/srep21424)
Supplement: Supplementary Information [file srep21424-s1.pdf]

Supplementary Information for "Quantum-mechanical machinery for rational decision-making in classical guessing game"

Jeongho Bang,[1,2,3,*] Junghee Ryu,[3] Marcin Pawłowski,[3] B. S. Ham,[1,†] and Jinhyoung Lee[2,‡]

[1]*Center for Photon Information Processing, and School of Information and Communications, Gwangju Institute of Science and Technology, Gwangju, Republic of Korea*

[2]*Department of Physics, Hanyang University, Seoul 133-791, Korea*

[3]*Institute of Theoretical Physics and Astrophysics, University of Gdańsk, 80-952 Gdańsk, Poland*

(Received December 31, 2015)

Abstract

Supplementary Information of "Quantum-mechanical machinery for rational decision-making in classical guessing game."

*Electronic address: jbang@gist.ac.kr

†Electronic address: bham@gist.ac.kr

‡Electronic address: hyoung@hanyang.ac.kr

I. 'WELL-QUANTIFIED' AND 'ILL-QUANTIFIED' PROBABILITIES OF BOB'S PREFERENCES

We give further analyses for well-quantified and ill-quantified probabilities of Bob's preferences. Firstly, we recall the total average score $\Xi_{\text{Bob}}^{(C)}$ of Bob achievable from the classical probabilistic reasoning. From Eq. (11) and Eq. (12) in our main text, we write $\overline{\xi}_{\text{Bob},\tau}^{(C)}$ ($\tau = 1, 2, 3, 4$) as below.

$$\begin{aligned}
\overline{\xi}_{\text{Bob},1}^{(C)} &= \alpha_0 + 2\alpha_0\alpha_1, \\
\overline{\xi}_{\text{Bob},2}^{(C)} &= \alpha_0 - 2\alpha_0\alpha_1, \\
\overline{\xi}_{\text{Bob},3}^{(C)} &= -\alpha_0 + 2\alpha_0\alpha_1, \\
\overline{\xi}_{\text{Bob},4}^{(C)} &= -\alpha_0 - 2\alpha_0\alpha_1.
\end{aligned} \tag{A1}$$

It is clear that if there is no bias among the preferences, i.e., $\alpha_0 = \alpha_1 = 0$, then $\overline{\xi}_{\text{Bob},\tau}^{(C)} = 0$ for all $\tau = 1, 2, 3, 4$. However, if Bob has $\alpha = (\alpha_0, \alpha_1)^T \neq (0, 0)^T$ for the given hints, Bob can improve his winning average with the appropriately assigned directional conditions of α (see Fig. 1 in our main text) . More specifically, Bob can have

$$\Xi_{\text{Bob,best}}^{(C)} = |\alpha_1| + 2\,|\alpha_1|\,|\alpha_2| > 0, \tag{A2}$$

as described in the main text [see Eq. (13)]. However, if the hinting is malicious, Bob may fail. To see this clearly, we draw the graphs of $\overline{\xi}_{\text{Bob},\tau}^{(C)}$ for the cases $\tau = 1, 2, 3, 4$ (see Fig. 1 in our main text). In each graph, we specify the regions of well-quantified (red dashed box) and ill-quantified (green dashed box) probabilities in the space of $(\alpha_0,\ \alpha_1)$. Here we can imagine any malicious hinting that misleads Bob toward the green dashed regions. This is the worst scenario for Bob, in which he will have the score

$$\Xi_{\text{Bob,worst}}^{(C)} = -\,|\alpha_1| - 2\,|\alpha_1|\,|\alpha_2|\,. \tag{A3}$$

If Bob has to take into account all these situations, it is evident that Bob will have $\Xi_{\text{Bob}} = 0$, because he cannot have any preferences for the given hints.

Then, turning our analysis to the quantum reasoning, let us write $\xi_{\text{Bob},\tau}^{(Q)}$, by using Eq. (11) and Eq. (14) in the main text, as

$$\overline{\xi}_{\text{Bob},1}^{(Q)} = \overline{\xi}_{\text{Bob},1}^{(C)} + \Gamma\cos(\pi\Delta),$$

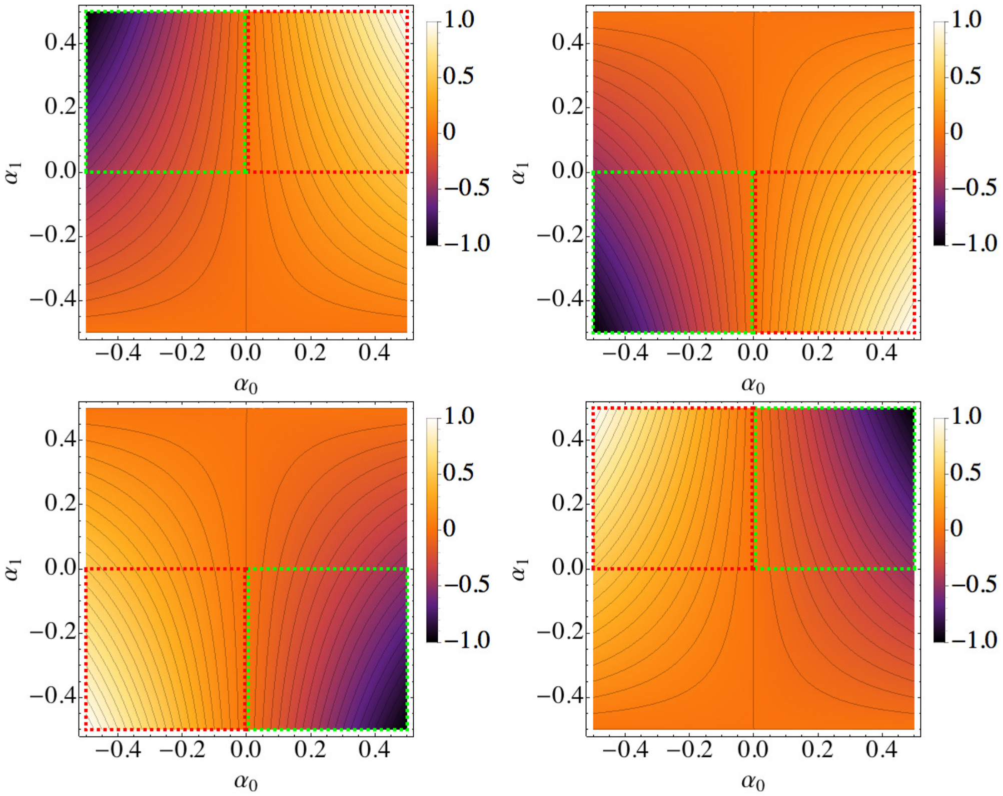

FIG. S1: We depict Bob's score $\overline{\xi}^{(C)}_{\text{Bob},\tau}$ averaged for a specific set of $u_{\text{Alie}}(x)$ ($x = 0, 1$): (top-left) [$\boldsymbol{\tau}$.**1**] (top-right) [$\boldsymbol{\tau}$.**2**], (bottom-left) [$\boldsymbol{\tau}$.**3**], and (bottom-right) [$\boldsymbol{\tau}$.**4**]. We specify the regions of 'good' (red dashed box) and 'bad' (green dashed box) probabilities (see, also, Fig. 1 in our main text).

$$\overline{\xi}^{(Q)}_{\text{Bob},2} = \overline{\xi}^{(C)}_{\text{Bob},2} - \Gamma \cos(\pi\Delta),$$
$$\overline{\xi}^{(Q)}_{\text{Bob},3} = \overline{\xi}^{(C)}_{\text{Bob},3} + \Gamma \cos(\pi\Delta),$$
$$\overline{\xi}^{(Q)}_{\text{Bob},4} = \overline{\xi}^{(C)}_{\text{Bob},4} - \Gamma \cos(\pi\Delta), \tag{A4}$$

where the Γ is given as [see Eq. (15) in our main text]

$$\Gamma = 2\sqrt{\left(\frac{1}{4} - |\alpha_1|^2\right)\left(\frac{1}{4} - |\alpha_2|^2\right)}. \tag{A5}$$

Here, it is also true that Bob cannot improve his winning average when $\alpha_0 = \alpha_1 = 0$. In such a case, Bob has $\overline{\xi}^{(C)}_{\text{Bob},\tau} = 0$ with $\cos(\pi\Delta) = 0$ for all $\tau = 1, 2, 3, 4$. However, if he can use well-quantified probabilities in his quantum reasoning, the average score can be higher than Eq. (A2). Specifically, Bob can have

$$\Xi^{(Q)}_{\text{Bob,best}} = \Xi^{(C)}_{\text{Bob,best}} + \Gamma, \tag{A6}$$

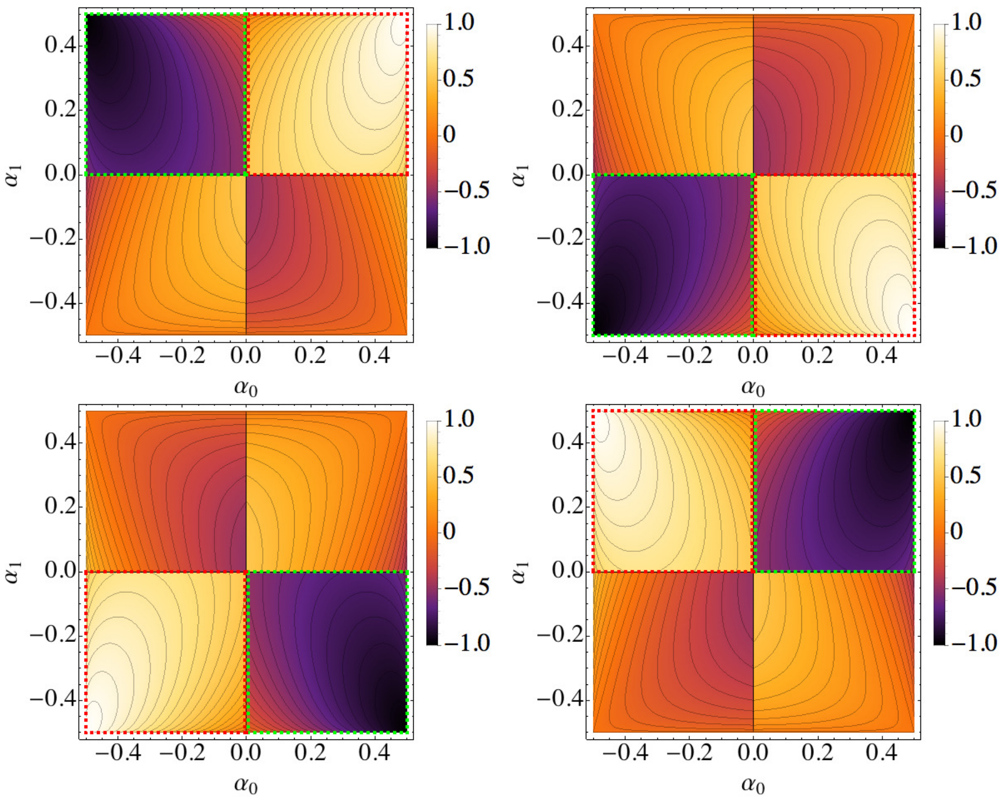

FIG. S2: We give the graphs of $\bar{\xi}^{(Q)}_{\mathrm{Bob},\tau}$ for (top-left) [$\boldsymbol{\tau}$.**1**], (top-right) [$\boldsymbol{\tau}$.**2**], (bottom-left) [$\boldsymbol{\tau}$.**3**], and (bottom-right) [$\boldsymbol{\tau}$.**4**]. We also specify the regions of 'good' (red dashed box) and 'bad' (green dashed box) probabilities.

as described in the main text [see Eq. (16)]. However, there can also be malicious hinting, in which case Bob may fail, similarly to the classical case. Let us see the graphs of $\bar{\xi}^{(Q)}_{\mathrm{Bob},\tau}$ in Fig. S2, where the regions of well-quantified (red dashed box) and ill-quantified (green dashed box) probabilities are also specified. From the same analysis as in the case of the classical probabilistic reasoning, we can see that Bob's total average scores can be decreased. Notably, in the worst case, such disadvantages can be maximized as

$$\Xi^{(Q)}_{\mathrm{Bob,worst}} = \Xi^{(C)}_{\mathrm{Bob,worst}} - \Gamma. \tag{A7}$$

This implies that the quantum reasoning can make the situation worse. In the case where Bob cannot evaluate whether the given hints are good or not, it is not possible for Bob to improve his winnings, as described above.

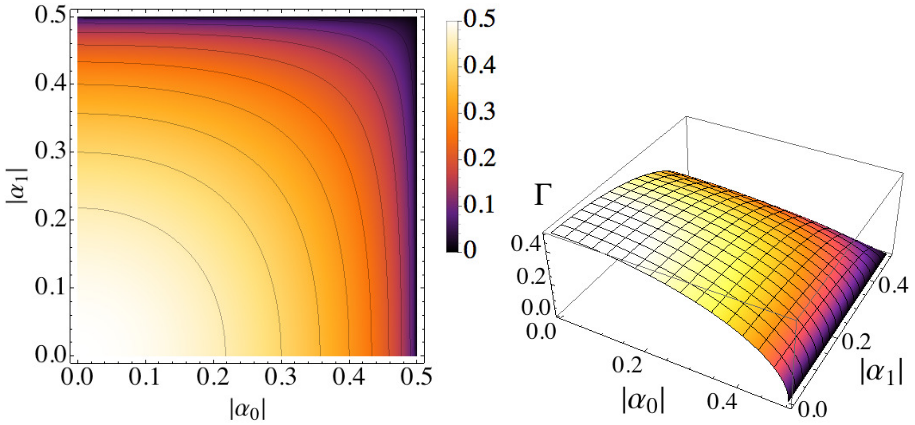

FIG. S3: The graphs of Γ (density-plot on the left, and 3D-plot on the right) with respect to $|\alpha_0|$ and $|\alpha_1|$. The probabilities of Bob's preferences are assumed to be well-quentified.

II. THE QUANTUM ADVANTAGE $\Gamma = \Xi_{\mathbf{Bob}}^{(Q)} - \Xi_{\mathbf{Bob}}^{(C)}$ AND THE AVERAGE SCORE $\Xi_{\mathbf{Bob}}^{(Q)}$ OF THE QUANTUM REASONING

Here we consider the quantum advantage, i.e., $\Gamma = \Xi_{\text{Bob}}^{(Q)} - \Xi_{\text{Bob}}^{(C)}$, and the average score $\Xi_{\text{Bob}}^{(Q)}$ of the quantum reasoning more carefully. To do this, we recall $\Gamma = 2\sqrt{\left(\frac{1}{4} - |\alpha_1|^2\right)\left(\frac{1}{4} - |\alpha_2|^2\right)}$ in Eq. (A5) and draw the graphs of Γ as in Fig. S3. Then we can make the following observation. The quantum advantage Γ is maximized when $|\alpha_j| \to 0$ $(j = 0, 1)$ [1] and is vanished when $|\alpha_0| = \frac{1}{2}$ or $|\alpha_1| = \frac{1}{2}$. With this observation, we find that, interestingly, the average score $\Xi_{\text{Bob}}^{(Q)}$ can be higher with the weak preferences than with the stronger ones (see Fig. S4). Thus one may consider a peculiar situation that, even though the preferences become stronger with additional 'good' hints, the average score $\Xi_{\text{Bob}}^{(Q)}$ could decrease. Of course, $\Xi_{\text{Bob}}^{(Q)}$ is still higher than $\Xi_{\text{Bob}}^{(C)}$ even in this case. However, such a situation cannot be considered as being natural in a realistic circumstance, as the hints are usually not independent but correlated with one another. In particular, it is unusual that, for a given hints, only one of $|\alpha_j|$ $(j = 0, 1)$ becomes close to the target whereas the other does not.

---

[1] Such a feature was also observed in our numerical simulation (see Fig. 8 in our main text).

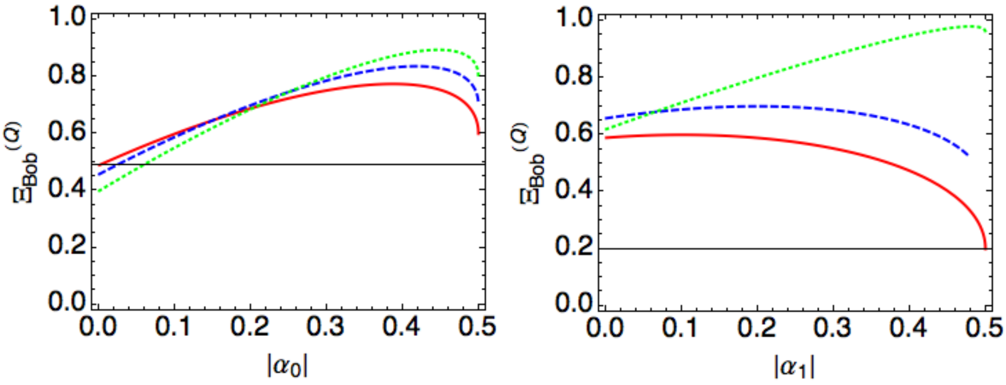

FIG. S4: The average score $\Xi_{\mathrm{Bob}}^{(Q)}$ of the quantum reasoning: On the left side, the graph of $\Xi_{\mathrm{Bob}}^{(Q)}$ versus $|\alpha_0|$ is given for $|\alpha_1| = 0.1$ (red solid), 0.2 (blue dashed), and 0.3 (green dotted). On the right side, the graph of $\Xi_{\mathrm{Bob}}^{(Q)}$ versus $|\alpha_1|$ is given for $|\alpha_0| = 0.1$ (red solid), 0.2 (blue dashed), and 0.3 (green dotted).
